# Nosocomial acquisition of influenza is associated with significant morbidity and mortality: Results of a prospective observational study

**DOI:** 10.1016/j.jiph.2022.08.021

**Published:** 2022-10

**Authors:** L.B. Snell, J.P. Vink, N.Q. Verlander, S. Miah, A. Lackenby, D. Williams, H. Mitchell, C. Beviz, M. Kabia, E. Cunningham, R. Batra, J.D. Edgeworth, M. Zambon, G. Nebbia

**Affiliations:** a Centre for Clinical Infection and Diagnostics Research, Guy’s and St. Thomas’ NHS Foundation Trust, London, UK; b Department of Infection, Guy’s and St Thomas’ NHS Foundation Trust, UK; c UK Health Security Agency, Colindale, UK; d Infection Sciences, Viapath, London, UK

**Keywords:** Influenza, Hospital-acquired infection, Infection prevention and control

## Abstract

**Background:**

Nosocomial acquisition of influenza is known to occur but the risk after exposure to a known case and the outcomes after acquisition are poorly defined.

**Methods:**

Prospective observational study of patients exposed to influenza from another patient in a multi-site healthcare organisation, with follow-up of 7 days or until discharge, and PCR-confirmation of symptomatic disease. Multivariable analysis was used to investigate association of influenza acquisition with high dependency unit/intensive care unit (HDU/ITU) admission and in-hospital mortality.

**Results:**

23/298 (7.7%) contacts of 11 cases were subsequently symptomatic and tested influenza-positive during follow-up. HDU/ITU admission was significantly higher in these secondary cases (6/23, 26%) compared to flu-negative contacts (20/275, 7.2%; p = 0.002). In-hospital mortality was significantly higher in secondary cases (5/23, 21.7%) compared to flu-negative contacts (11/275, 4%; p < 0.001). In multivariable analysis, age (OR 1.25 95% CI: 1.01–1.54, p = 0.02) and being a secondary case (OR 4.77, 95% CI: 1.63–13.9, p = 0.008) were significantly associated with HDU/ITU admission in contacts. Age (OR 1.00, 95% CI: 0.93–1.00, p = 0.02), being a secondary case after exposure to influenza (OR 3.81, 95% CI 1.09–13.3, p = 0.049) and co-morbidity (OR 1.29 per unit increment in the Charlson score, 95% CI 1.02–1.61, p = 0.03) were significantly associated with in-hospital mortality in contacts.

**Conclusions:**

Nosocomial acquisition of influenza was significantly associated with increased risk of HDU/ITU admission and in-hospital mortality.

## Background

Influenza causes annual outbreaks across the world and prior to the emergence of SARS-CoV-2 was the largest infectious disease killer in Europe [1]. In the UK alone, excess influenza deaths are modelled to exceed 20,000 during some seasons [2], around 95% of which occur in those aged over 65 [3]. In one Swiss region, 0.5% of all admissions received a diagnosis of nosocomial influenza [4].

Most data on the risk posed from nosocomial acquisition of influenza comes from studies in long term care facilities, where attack rates after exposure are reported to be between 20% and 90%, and with an associated mortality from 5% to 50% [5], [6], [7], [8], [9], [10], [11], [12]. Patients who acquire nosocomial influenza were more likely to be older and immunosuppressed in one retrospective cohort study [13]. Immunosuppression was also associated with acquisition in another retrospective multivariable analysis [14].

There is little data on whether the associated mortality of nosocomial influenza acquisition is attributable to infection or instead being similar to other individuals of similar age and comorbidity. One large study in Canada found no difference in mortality between community and nosocomial influenza, but failed to account for differences in morbidity [15].

We designed a prospective observational study to address these issues. We focussed on those inpatients exposed to influenza from other patient cases, referred to as ‘contacts’. Here we present data to look at the attack rate of nosocomial exposure and the outcomes for these exposed individuals who acquire symptomatic infection. In this analysis we control for confounding variables, such as age and comorbidity, to look at the risk posed by nosocomial acquisition. Finally, we provide genome sequencing data to investigate these putative transmission networks.

## Methods

### Ethical approval

The protocol was approved by the Trust’s Clinical Governance and Clinical Audit department (reference number 10514). Informed consent was not required for inclusion as the data used was collected by the direct care team as part of routine clinical care. In addition, there was no change in patient management or infection control policy.

### Identification of index cases and their exposed contacts

Adult patients (over 18 years old) attending our hospitals between December 2018 and February 2019 with a laboratory-confirmed diagnosis of influenza were identified from daily lab reports. Viral testing of upper respiratory tract swabs was completed using either Aus Diagnostic multiplex PCR or Cepheid Xpert Flu as per our standard hospital testing pathways.

Cases of interest (‘cases’) were adult patients with a laboratory confirmed diagnosis of influenza attending the Emergency Department, one of our Assessment Units (e.g. medical, antenatal, oncology), or diagnosed whilst an inpatient. We then identified ‘contacts’, defined as those patients who were exposed to infection by these influenza cases. Exposure was defined as sharing a bay/room with a laboratory-confirmed case at the time of testing, in the absence of the isolation precautions that would be expected for suspected or confirmed cases of influenza as per standard infection control protocols. Contacts were considered exposed to influenza if they spent any length of time in the same bay as a case. The median turnaround time between sample collection and influenza result was 1 day and 10 h during this study; as such all contacts identified after a positive result were considered to have had sufficient exposure to be considered contacts, and sufficient incubation time to allow day 1 follow-up after exposure to occur upon the test result of the case.

The contacts were then followed up for 7 days during their admission or until their discharge (whichever came first) to determine if they became symptomatic, at which point they were tested for influenza as per hospital policy. Symptoms could include any upper respiratory tract illness, respiratory deterioration or unexplained fever. A contact that had a first influenza-positive swab after being identified as a contact is therefore referred to as a ‘secondary case.’ Contacts in whom a laboratory confirmed diagnosis of influenza was not made are referred to ‘flu-negative contacts’.

Cases and contacts were entered into a database to document details of their diagnosis including date and time of diagnosis, ward, basic demographic details, smoking history, pregnancy status and co-morbidities. Data on co-morbidities was collected to produce a Charlson co-morbidity index for each case and contact. Smoking was categorised as either current smoker, ex-smoker, non smoker or unknown. Other information collated included details of sample timings, length of stay, admission to high dependency or intensive care wards (HDU/ITU), and in-hospital mortality.

### Infection control

Pre-existing infection control policy for our hospitals were followed during the study period. In brief, patients with confirmed or suspected influenza are isolated in side rooms or cohort bays/rooms with other cases. After a confirmed case of influenza, particularly one which was not previously suspected, the case is moved to a side room or cohort bay/room with other confirmed cases. Other patients in the same bay when the case is identified are assumed to be exposed to influenza (i.e. contacts) and are isolated to prevent onward transmission. New admissions to the bay are stopped. These contacts were followed up and monitored for symptoms as described above.

### Statistical analysis

Statistical analysis including single variable and multivariable logistic regression completed using Stata v15.1. Single variable regression was used to examine associations between risk factors and in-hospital mortality, determining odds ratios (OR), significance (p) values and 95% confidence intervals (CI). Risk factors included in single variable analysis for in-hospital death, including age, gender, smoking status, oseltamavir prophylaxis and treatment use, pregnancy, status as a secondary case, length of stay prior to becoming a contact and Charlson co-morbidity index. For smoking history regression was conducted with a reference group of ‘current smoker’ against a group containing any other smoking status. Vaccination status was unavailable in 208/298 (70%) of contacts, so was not included in analysis.

Those risk factors from single variable analysis with raised odds ratio (OR) and p-value less than 0.2, as ascertained by the likelihood ratio test (LRT), together with age and sex as potential confounders, were included in an initial backwards stepwise multivariable model. Variables were removed one at a time, with protective factors first, until all variables had p-value from LRT less than or equal to 0.1 or were substantial confounders. A substantial confounder was one for which its removal resulted in a change in the OR of at least twenty percent in one or more of the parameters still in the model. The most appropriate polynomial form on the logit scale for a continuous variable and the outcome was chosen among the class of cubic, quadratic and linear functions, by LRT and using 5% as the significance level. Variables were then removed one at a time if p-value was larger than 0.1 and not substantially confounding, in decreasing order of p-value.

### Whole genome sequencing and phylogenetic analysis of residual influenza isolates

Whole genome sequencing from residual nose and throat swabs was carried out using short-read Illumina technology on MiSeq platform. Sequenced Isolates were included in analysis if the hemagglutinin gene was sequenced with mapped read depth > =10 and genome coverage > = 85%.

Phylogenetic trees were produced by the reference laboratory based on analysis of the hemagglutinin gene. To estimate genetic distance we considered an upper limit of the rate of molecular evolution of influenza A as 0.006 subs/site/year and the longest duration between a directly transmitting and receiving pair of patients to be 7 days. Given the number of sites used to reconstruct the phylogenies, we estimated the highest plausible quantity of substitutions we would expect between a transmission pair, arriving at a threshold of two permitted substitutions within each cluster. Phylogenies were inferred for each haemagluttinin subtype.

## Results

### Influenza cases and their contacts

**Fig. 1:**
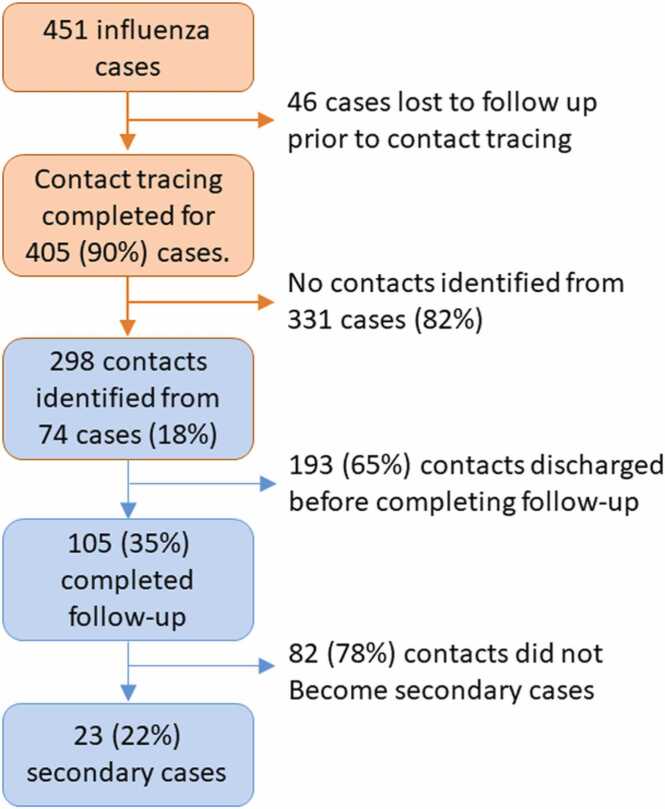
Flow chart of influenza cases and contacts identified during the study period.

Fig. 1 details a flow chart of influenza cases and contacts. 405 patients met our case definition and were included in the database. This represents 405/451 (90%) of eligible cases, as decided by retrospective querying of the laboratory database. Influenza A represented 404/405 (99.8%) of our cases.

Contacts were identified from 74/405 (18.3%) of cases. A total number of 298 contacts were identified (range 1–22 per case, median 3). The mean age of contacts was 63 years old (range 18–98) and 165/298 (55.4%) were female.

### Follow-up of contacts, transmission events and outcomes

The number of contacts present until the end of their 7-day follow-up was 105/298 (35.2%). The median length of follow-up after being identified as a contact before discharge or death was 5.0 days.

23/298 (7.7%) of all contacts, or 23/105 (21.9%) of contacts that completed follow-up, from 11 cases were subsequently symptomatic and tested influenza-positive during follow-up, and thus designated as secondary cases. From these 11 cases which resulted in secondary cases, there was a median of 1 secondary case detected (range 1–5).

Table 1 compares the demographics of flu secondary cases verses flu-negative contacts.

**Table 1:** Demographics of contacts subdivided to whether they subsequently tested positive and became a secondary case or remained negative (‘flu-negative contacts).

	Secondary cases (n = 23)	Flu-negative contacts (n = 275)
Gender		
Male	15 (65%)	150 (55%)
Female	8 (35%)	125 (45%)
Age		
< 50	2 (9%)	76 (28%)
50–59	4 (17%)	40 (15%)
60–69	4 (17%)	46 (17%)
70–79	3 (13%)	61 (22%)
≥ 80	10 (43%)	52 (19%)
Co-morbidities		
Mean Charlson score	5.6	3.7
Immunosuppressed	3 (13%)	33 (12%)
Current smoker	4 (17%)	47 (17%)
Pregnant	1 (4%)	13 (5%)
Current alcohol excess	0 (0%)	17 (6%)
Prophylaxis use	15 (65%)	160 (58%)

In total there were 26/298 (8.7%) admissions to HDU/ITU and 16/298 (5.4%) in-hospital deaths in the group of contacts. HDU/ITU admission was significantly higher in secondary cases (6/23, 26%) compared to flu-negative contacts (20/275, 7.2%; p = 0.002). In-hospital mortality was significantly higher in secondary cases (5/23, 21.7%) compared to contacts who remained flu-negative (11/275, 4%; p < 0.001).

### Genome sequencing of influenza to investigate putative transmission

**Fig. 2:**
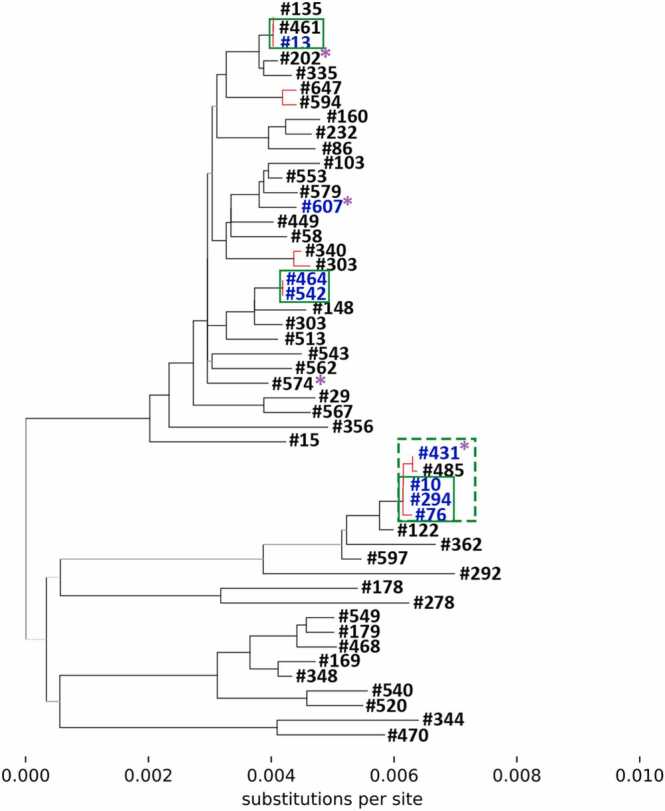
Maximum likelihood phylogenetic tree of whole genome sequences of influenza of H1N1 subtype from residual samples. Branches are labelled with anonymous patient identification number. Branches where viral genomes show high relatedness are coloured in red. Cases use black font colour; secondary cases blue font colour. Cases or secondary cases which are the only member of their prospectively identified epidemiological clusters represented on the phylogeny are marked with a purple asterisk. Cases and secondary cases linked by both epidemiological and genomic analysis are grouped with a green box. Where the box has a dashed outline this represents cryptic transmission.

**Fig. 3:**
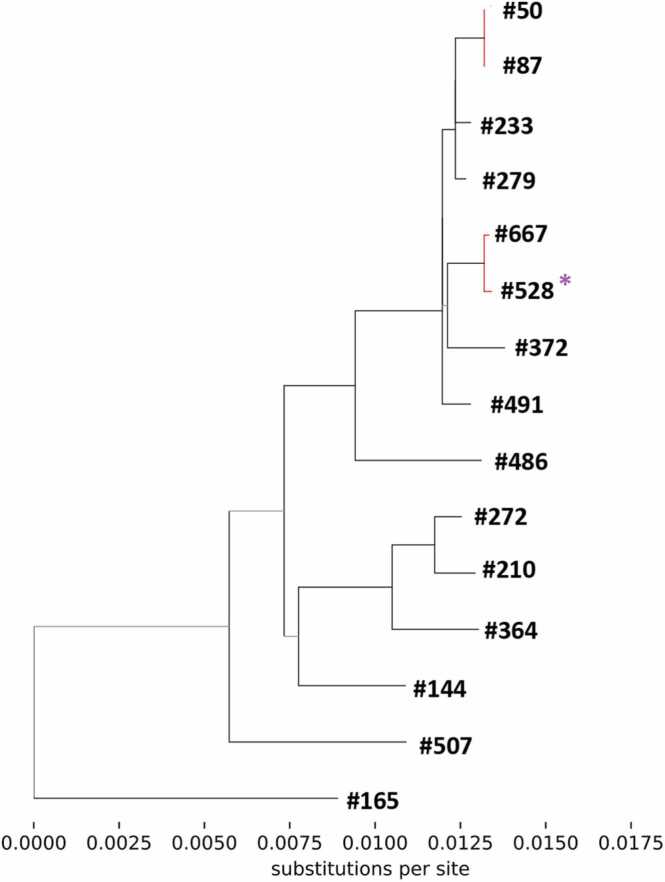
Maximum likelihood phylogenetic tree of whole genome sequences of influenza of H3N2 subtype from residual samples. Branches are labelled with anonymous patient identification number. Branches where viral genomes show high relatedness are coloured in red. Cases use black font colour; secondary cases blue font colour. Cases or secondary cases which are the only member of their prospectively identified epidemiological clusters represented on the phylogeny are marked with a purple asterisk. Cases and secondary cases linked by both epidemiological and genomic analysis are grouped with a green box.

126 residual samples from cases and secondary cases were retrieved from the diagnostic laboratory for whole genome sequencing. 65 cases yielded sequence with sufficient coverage and depth for further phylogenetic analysis (Fig. 2 & 3). This included 12 cases and secondary cases, from 8/11 of our prospectively identified clusters.

Three (3/8) clusters had more than one case or secondary case represented on the phylogenetic analysis, including 7/12 influenza-positive patients in total. All seven of these patients co-segregated with other influenza-positive patients from their clusters as defined by initial epidemiological analysis: case #461 with secondary case #13; secondary cases #464 and #542 exposed by the same case; and secondary cases #10, #294, and #76 exposed by the same case. One other case (#485) for whom no contacts were prospectively identified and therefore was not part of a prospectively identified cluster, also co-segregated with one (1/12) secondary case (#431) from another cluster (1/8), and three secondary cases from another cluster already described (#10, #294, and #76). Further epidemiological investigation showed that they had overlapping ward stays within one week prior to diagnosis, suggesting a cryptic transmission network that was not detected by initial epidemiologic investigation. The 4/12 remaining patients from 4/8 other clusters were the only patients from their cluster represented on the phylogeny (#607, #202, #574; #528).

Importantly, 4 pairs of cases (#667 and #528; #50 and #87; #647 and #594; #340 and #303) had viral genomes with sufficient relatedness to suggest linkage, however epidemiological analysis showed no temporal or spatial overlap within the hospital, suggesting the genomic linkage was spurious and did not represent a transmission event.

### Single variable analysis of factors associated with HDU/ITU admission and in-hospital mortality of contacts

Risk factors for HDU/ITU admission in contacts were investigated using single variable analysis (Table 2). Influenza acquisition (i.e. being a secondary case) gave an OR of 6.67 (CI 2.09–21.3, p = 0.006) for HDU/ITU admission. Age and Charlson score were also found to be risk factors for HDU/ITU admission, with OR of 1.03 per year older (CI 0.98–1.03, p = 0.049) and 1.41 per unit increase of Charlson score (CI 1.15–1.72, p = 0.0006). Gender, length of stay prior to exposure, pregnancy, smoking history and immunosuppression were not significantly associated with HDU/ITU admission of contacts in single variable analysis.

**Table 2:** Univariable analysis of factors associated with HDU/ITU admission of contacts.

	OR	95% CI	LRT p
Secondary case	No1.0 Yes6.67	2.09–21.3	0.0056
Gender (Female)	2.53	0.80–8.03	0.0629
Age	1.03 per year	1.00–1.06	0.0489
Length of stay before exposure	1.01 per day	0.98–1.03	0.6920
Charlson score	1.41 per unit increase	1.15–1.72	0.0006
Pregnancy	10^−7^		0.1129
Current smoker	1.13	0.31–4.10	0.9523
Immunosuppressed	1.74	0.47–6.43	0.4918

Risk factors for in-hospital mortality in contacts were investigated using single variable analysis (Table 3). Influenza acquisition in contacts (i.e being a secondary case) was found to be a risk factor for in-hospital mortality with an OR of 4.5 (CI 1.60–12.7, p = 0.0094). Gender, age, length of stay prior to exposure, Charlson score, smoking status and immunosuppression were not significantly associated with in-hospital mortality of contacts in single variable analysis.

**Table 3:** Univariable analysis of factors associated with in-hospital mortality of contacts.

	OR	95% CI	LRT p
Secondary case	No1.0 Yes 4.5	1.60–12.7	0.0094
Gender (Female)	1.27	0.56–1.83	0.565
Age	1.01 per year	0.98–1.03	0.5975
Length of stay prior to exposure	1.01	0.99–1.02	0.5115
Charlson score	1.10 per unit increase	0.98–1.27	0.1893
Current smoker	0.38	0.09–1.66	0.1452
Immunosuppressed	0.94	0.27–3.32	0.9288

### Multivariable analysis of factors for HDU/ITU admission and in-hospital mortality in contacts

Table 4 shows the results of multivariable analysis for HDU/ITU admission. There was a significant association between age and HDU/ITU admission. Being a secondary case was a risk factor for HDU/ITU admission with an estimated OR of 4.77 (CI 1.63–13.9, p = 0.008). Neither Charlson score or gender were a significantly associated with HDU/ITU admission.

**Table 4:** Final multivariable model of factors associated with HDU/ITU admission of contacts.

Variable	Categorisation	OR (95% CI)	p-value
Age		Linear: 1.25 (1.01–1.54) Quadratic: 1.00 (1.00–1.00)	0.02
Secondary case	No Yes	1.00 4.77 (1.63–13.9)	0.008

Table 5 shows the results of multivariable analysis for in-hospital mortality in contacts. There was a significant non-linear (cubic) association between age and outcome (p = 0.02). The Charlson score was significantly associated with in-hospital mortality with an OR of 1.29 (95% CI: 1.02, 1.61, p = 0.03) for every additional point. Being a secondary case was also associated with in-hospital mortality, with a OR of 3.81 (1.09–13.3 p = 0.049). Gender was a substantial confounder for the mortality outcome only (p = 0.16).

**Table 5:** Final multivariable model of factors associated with in-hospital mortality of contacts.

Variable	Categorisation	OR (95% CI)	p-value
Age		Cubic: 1.00 (0.93–1.00)	0.02
Gender	Male Female	1.00 0.42 (0.11–1.53)	0.16
Secondary case	No Yes	1.00 3.81 (1.09–13.3)	0.049
Charlson score (not including age)		1.29 per unit increment (1.02–1.61)	0.03

## Discussion

Our study suggests a significant increase in HDU/ITU admission and in-hospital mortality in patients exposed to influenza whilst in hospital who subsequently develop symptomatic disease. This effect persists even when controlling for age, length of stay and co-morbidities, with acquisition of influenza given an odds ratio of around 5-fold for HDU/ITU admission and 4-fold for in-hospital mortality. In addition, age and Charlson score were a strong risk factor in-hospital mortality. Identification of these factors that are known to be associated with poorer outcomes for inpatients increases confidence in our findings. To our knowledge this is the first time a prospective study has sought to estimate the risk of hospital acquisition of influenza on mortality. As the exposure events for these contacts are known, and given they were asymptomatic at time of enrolment, this increases confidence these are genuine nosocomial acquisitions.

One previous retrospective study found nosocomial acquisition of influenza was associated with approximately three times the risk of complications, including septic shock, ventilation and death [16]. Another retrospective study found influenza acquisition in hospital showed a trend to significant association with death when adjusted for age and Charlson score [17]. Our results add to this evidence. Our findings have important generalisability in the UK and the European Union, where patients sharing rooms is a frequent occurrence. Such shared spaces risks transmission of influenza between patients. However, in settings where patients are nursed in single rooms the risk of transmission between patients is likely to be less.

Genome sequencing of viral isolates did not refute any of the putative transmission clusters, providing some support for three of the clusters identified epidemiologically. In addition, genetic similarity between cases successfully sequenced suggested cryptic transmission. Some of these cases were epidemiologically linked retrospectively, despite not having been identified prospectively. This cryptic transmission has been identified by other studies and could involve ‘missing links’ not identified by our contact tracing. We also found genomic similarity between some cases where no epidemiological linkage was evident, which concurs with a previous study which found only limited evidence of epidemiological overlap between genomically clustered cases [18]. Other studies have used whole genome sequencing to refute epidemiologically linked cases where cases do not share sufficient genomic similarity [19], [20].

Our study shows the pitfalls of using residual sample for outbreak investigation. Only around one quarter of samples had sufficient residual extract available for sequencing. Out of these, sequence of sufficient quality was only obtained for 12 of our cases and secondary cases. For this reason, we were unable to use genomics to investigate the majority of our putative transmissions. Since improving our storage processes we have successfully investigated outbreaks from other viral pathogens, for instance SARS-CoV-2 [21].

This study likely underestimates the number of contacts who became infected after exposure. Firstly, more than half of contacts did not complete follow-up, as their discharge occurred prior to this time point. In addition, asymptomatic cases will be missed as our study design followed hospital infection control policy, which does not include swabbing of asymptomatic individuals. Exposure was also limited to those patients who shared a bay with cases, and is thus likely to underestimate the number of contacts, such as healthcare workers and patient contacts in other areas. Patient tracking should be considered to better address this issue, such as live bed state not available in our hospitals.

The risk of mortality seen here with hospital acquisition of influenza supports the use of post exposure prophylaxis in individuals judged at risk of severe influenza. Currently, national prophylaxis guidelines do recommend use of post exposure prophylaxis in those over the age of 65 and with predisposing conditions such as diabetes, neurological, hepatic, renal, pulmonary and chronic cardiac disease [22]. Further work could include assessment of the effect of vaccination, which could not be included here due to poor data availability, as well as prophylaxis and treatment on outcomes after hospital-acquisition of influenza.

## Funding

This project was funded by Guy’s and St Thomas’ Charity (https://www.gsttcharity.org.uk/; TR130505). The funders had no role in study design, data collection, analysis or otherwise. LBS and GN receive funding from the Medical Research Council (MR/W025140/1; MR/T005416/1)

## Conflicts of interest

None.
